# A standard ballroom and Latin dance program to improve fitness and adherence to physical activity in individuals with type 2 diabetes and in obesity

**DOI:** 10.1186/1758-5996-6-74

**Published:** 2014-06-22

**Authors:** Felice Mangeri, Luca Montesi, Gabriele Forlani, Riccardo Dalle Grave, Giulio Marchesini

**Affiliations:** 1 Unit of Endocrinology and Diabetology, General Hospital, Gavardo, Brescia, Italy; 2 Unit of Metabolic Diseases and Clinical Dietetics, “Alma Mater Studiorum” University of Bologna, Policlinico S. Orsola, Via Massarenti, 9 I-40138, Bologna, Italy; 3 Department of Eating and Weight Disorders, Villa Garda Hospital, Garda, Italy

## Abstract

**Objective:**

To test the effectiveness of a dance program to improve fitness and adherence to physical activity in subjects with type 2 diabetes and obesity.

**Research Design and Methods:**

Following a motivational interviewing session, 100 subjects with diabetes and/or obesity were enrolled either in a dance program (DP, n = 42) or in a self-selected physical activity program (SSP, n = 58), according to their preferences. Outcome measures were reduced BMI/waist circumference, improved metabolic control in type 2 diabetes (−0.3% reduction of HbA1c) and improved fitness (activity expenditure >10 MET-hour/week; 10% increase in 6-min walk test (6MWT)). Target achievement was tested at 3 and 6 months, after adjustment for baseline data (propensity score).

**Results:**

Attrition was lower in DP. Both programs significantly decreased body weight (on average, −2.6 kg; P < 0.001) and waist circumference (DP, −3.2 cm; SSP, −2.2; P < 0.01) at 3 months, and the results were maintained at 6 months. In DP, the activity-related energy expenditure averaged 13.5 ± 1.8 MET-hour/week in the first three months and 14.1 ± 3.0 in the second three-month period. In SSP, activity energy expenditure was higher but highly variable in the first three-month period (16.5 ± 13.9 MET-hour/week), and decreased in the following three months (14.2 ± 12.3; P vs. first period < 0.001). At three months, no differences in target achievement were observed between groups. After six months the odds to attain the MET, 6MWT and A1c targets were all significantly associated with DP.

**Conclusion:**

Dance may be an effective strategy to implement physical activity in motivated subjects with type 2 diabetes or obesity *(Clinical trial reg. no.*NCT02021890*, clinicaltrials.gov*).

## Introduction

Lifestyle changes aiming at healthy diet and habitual physical activity are mandatory for the prevention and treatment of type 2 diabetes [1], as well as in other metabolic disorders [2]. Dietary restrictions and physical activity promote weight loss, reduce the progression from prediabetes to diabetes [3], improve metabolic control and reduce the long-term risk of complications, at any stage of disease severity. Most guidelines recommend 150 min/week of moderate intensity physical activity, corresponding to 10–15 MET-hour/week and no more than 2 consecutive days without exercise [1,4]. Unfortunately, most adult individuals with metabolic disorders lead a sedentary life [5], and increasing physical activity is particularly challenging for overweight people due to aversion and poor tolerance of exercise [6].

Behavior changes are driven by personal motivation and stage of change to healthier lifestyles [7], and an increased intrinsic motivation for physical activity remains the strongest predictor of long-term results [8]. Specific programs to increase motivation and adherence to physical activity have been developed in subjects with type 2 diabetes [9], following the seminal experience of the Diabetes Prevention Program [10]. Both the metabolic control and the cardiovascular risk profile improve in parallel with the increased participation in structured physical activity programs [11,12]. Nonetheless, procedures and strategies derived from motivational interviewing and cognitive-behavior therapy are rarely applied outside research settings, and most patients continue to receive suboptimal or ineffective treatments.

Exercise adherence tends to increase when patients are free to select their own program and less structure is imposed [13]. The adoption of pleasant programs of leisure-time physical activity may be a reasonable option to increase motivation and adherence [14], while maintaining a supervised control on the amount of physical activity. Among leisure-time activities, dancing has a remarkable place [15]. When arranged by patients’ associations, aerobic dance, Latin dance, folk dance and standard ballroom dance may all help aging people to enjoy their physical activity. Dance exercise also stimulates positive emotions, promotes social interaction and creates relationships with other people in the group, while exchanging experiences about their common medical problems. Finally, the acoustic stimulation and the music might strengthen the beneficial effects of aerobic exercise on cognitive functions [16].

We aimed to test the metabolic and clinical effects of a 6-month program of dancing in subjects with type 2 diabetes and/or obesity, chronically cared for in two diabetes/metabolic units. Self-selection of the leisure time activity program was allowed to increase adherence to the physical activity programs.

## Materials and methods

### Study planning

BALLANDO (**B**enefici dell’**A**ttività fisica **L**udica sui **L**ivelli di **A**1c e grasso viscerale **N**el **D**iabete e **O**besità, i.e., beneficial effects of leisure-time physical activity on A1c levels and visceral body fat in diabetes and obesity) is a pilot study carried out in two diabetes/obesity units from January 2012 to March 2013, with the support of patients’ and sports associations. During a recruitment session carried out according to the principles of motivational interviewing [17], the patients were asked to select two different physical activity options: a) a standard ballroom and Latin dance program (DP) organized twice a week by diabetes associations, or b) a self-selected program (SSAP), with the option to receive support from sport associations, for training sessions to be carried out at least twice weekly. These patients were invited to register any bout of physical activity ≥30 min in a diary; the attendance of subjects to the dance sessions was systematically registered, not any additional physical activity exceeding the dancing program.

Inclusion criteria were age 40 to 70, type 2 diabetes in good-to-fair metabolic control (glycosylated hemoglobin (HbA1c) < 8.5%) and/or obesity (Body Mass Index (BMI) ≥ 30 kg/m^2^ or waist circumference >94 cm in males, >80 in females), with/without prediabetes or minor alterations of HbA1c. Exclusion criteria were previous cardiovascular events, muscle-skeletal problems reducing physical ability, any condition limiting systematic adherence.

Primary end-points were a 5% reduction in BMI or waist circumference, and improved energy expenditure (physical activity expenditure >10 MET-hour/week). Secondary outcomes were improved metabolic control in diabetes (0.3% reduction of HbA1c) and increased physical fitness (10% increase in the 6-min walk test - 6MWT). The target of 10 MET-hour/week corresponds to approximately 150 min fast/very fast walking, as from the Diabetes Prevention Program target [10], Italian Standards of diabetes care [18], and our own lifestyle modification programs [19]. The end-points were tested at 3- and 6-month follow-up.

The whole study was conducted in two diabetes/obesity units. All patients signed an informed consent. The protocol was approved by the ethical committee of Bologna University (Protocol #105/2011/U/Sper) (*Clinical trial reg. no.NCT02021890, clinicaltrials.gov*).

### Patients

The study enrolled 100 subjects (47 with type 2 diabetes, 53 with obesity); in 11 subjects with obesity, prediabetes was also present. Fifty-two were males, 48 females, mean age 59 [SD 9 (range, 41–70)]. Their complete demographic and clinical data are reported in Table 1. Most cases were enrolled in the Gavardo Unit, with only 11 patients recruited in Bologna. All subjects were followed in a continuous care model by the recruiting units, and had received counseling for healthy diet and habitual physical activity, but had not engaged in specific activity programs during the previous year. During the study, their contact with physicians was similar; both groups attended the specialist Units for ambulatory visits every 3 months, according to pre-specified protocols.

**Table 1:** Demographic and clinical variables in the population, grouped according to the selected activity program

**Demographic and clinical variables**	**Dance program (n = 42)**	**Self-selected program (n = 58)**	**P value**
Age (years)	58.5 ± 8.8	59.4 ± 8.5	0.611
Gender (M/F) (%)	43/57	59/41	0.156
Education (Primary/Secondary/Vocational/Degree) (%)	26/45/24/5	17/47/24/12	0.478
Occupation (Student/Employee/Self-employed/Retired/Other) (%)	0/21/19/33/26	7/28/26/34/5	0.022
Smoking (No/Yes/Ex) (%)	81/7/12	79/5/16	0.821
Alcohol (No/Yes/Ex) (%)	95/3/2	88/5/7	0.364
BMI class (Normal-weight/Overweight/Obesity) (%)	7/24/69	2/14/84	0.134
Glucose regulation (Normal/Prediabetes/Diabetes (%)	45/14/41	40/8/52	0.462

The reasons for program selection were largely dependent on personal interests, on preferences for group or individual programs, or finally on familiar or job constraints, making it difficult to adhere to a pre-specified time schedule. These differences were discussed during motivational interviewing [17], to stimulate adherence to the selected program. No patient was allowed to move from one program to the other after final assignment.

### Methods

DP consisted of a two-hour dancing session twice a week, led by instructors in a private hall of a disco. Both solo and partner and group dances were performed during each session: an initial one-hour activity, chaired by two instructors who taught new steps and choreographies to patients (both individually and in group), was followed by one hour dancing in pairs (Latin and standard ballroom music). Patients were free to dance with other participants or with their own partners, thus reinforcing family ties and friendship. During the initial four weeks, patients were monitored by health care personnel (nurses and physician); blood pressure, heart rate, fingertip glucose (in diabetes) were checked before and immediately after sessions.

SSAP consisted of a wide variety of activities. The preferred activities were walking (36 cases), cycling (4 cases), swimming (6 cases), gym sessions (5 cases - with the support of sports associations), or home exercising (exercise bike, 6 cases), but also included bouts of mountain walking, golf, weight lifting, jogging, dancing. Such activities were recorded in a diary, also reporting time, duration and average speed, without external confirmation.

The energy expenditure (in MET-hour per week) was calculated using the 2011 Compendium of Physical Activities [20], on the basis of the adherence to the programmed dance sessions (number of attended weekly sessions), the type of dance (MET, 4–7), as well as the recorded self-selected physical activity bouts (from MET 2.8 (walking, 3 km/hour) to MET 8.5 (aerobic step), according to average speed and perceived fatigue).

Anthropometric and clinical data, metabolic profile (routine biochemistry) and physical fitness were tested at baseline and after 3 and 6 months. Weight and height were measured using a calibrated scale and stadiometer to the nearest 0 · 5 kg and 0 · 5 cm; waist circumference was measured by a tape meter. The 6MWT was carried out as described by Enright [21].

At baseline, all subjects were tested for motivation to habitual physical activity by the EMME-3 test [22], as previously reported [23,24]. The test consists of two parts: a) an 18-item questionnaire (MAC 2) on a Likert scale from 0 (totally false) to 6 (totally true); b) a set of 6 visual analogue scales (VAS) from 0 to 100. Briefly, the test evaluates motivation-to-change according to Prochaska’s stage of change model (Precontemplation, Contemplation, Determination, Action, Maintenance) [25], and other psychological factors associated with motivation (Discrepancy, Self-Efficacy, Importance, Temptation, Readiness-to-change and Stabilization-of-change) [26,27].

### Statistical analysis

A descriptive analysis of data was carried out in the whole population and in different subgroups (e.g., with/without type 2 diabetes; according to activity programs). Their time course in response to the activity program was tested by repeated measures ANOVA.

To adjust results for baseline differences between groups, a propensity score approach was used [28]. The propensity score for the two activity programs was calculated by logistic regression on clinical, demographic and psychological variables at baseline that constitute potential barriers to group treatment, i.e., age, sex, educational level, occupation. The probability to reach the planned targets was tested by logistics regression in different models, having the primary targets as dependent variables and the type of program (DP vs. SSAP) as independent variable, after adjustment for propensity.

Four sets of variables were simultaneously tested (anthropometric, psychological, clinical and activity variables), and the significance limit was then adjusted to P = 1- ${}^{\left(n-1\right)}\sqrt{\left(1-p\right)}$ , where p = 0.05 and n = 4 [29]. The final critical value was therefore set at 0.015.

## Results

The participants covered a wide range of BMI (only 9% normalweight) and age (41–70). Thirty-four cases were 65 or over, mainly in the diabetes group (71%). No differences were recorded in education levels, smoking and alcohol intake between groups (Table 1). Also the psychological profile of motivation to habitual physical activity was similar and characterized by high scores of contemplation and determination, low values of precontemplation and action, and high rates of importance, self-efficacy and readiness-to-change (Table 2).

**Table 2:** Stage of change and psychological factors associated with motivation to habitual physical activity in patients grouped according to the selected activity program

**Psychological variables**	**Dance program (n = 42)**	**Self-selected program (n = 58)**	**P value**
Pre-contemplation (%)	24.2 ± 29.6	26.7 ± 20.0	0.626
Contemplation (%)	67.7 ± 23.1	64.8 ± 16.9	0.483
Determination (%)	80.4 ± 18.4	73.7 ± 21.2	0.113
Action (%)	39.1 ± 29.9	47.0 ± 27.3	0.183
Maintenance (%)	47.0 ± 32.3	41.0 ± 28.5	0.340
Discrepancy (%)	48.6 ± 25.6	52.5 ± 21.2	0.419
Importance (%)	82.6 ± 14.7	81.6 ± 14.4	0.745
Self-efficacy (%)	77.9 ± 15.0	70.7 ± 16.5	0.031
Temptation (%)	31.2 ± 24.8	42.1 ± 22.6	0.027
Readiness-to-change (%)	75.5 ± 21.9	73.3 ± 20.4	0.613
Stabilization-of-change (%)	61.8 ± 21.5	59.4 ± 25.9	0.665

All scores are reported mean ± SD in% of maximum score.

DP and SPP had similar rates of obesity, but the average BMI was over 2.5 points larger in SSAP (Table 3). Biochemistry and physical fitness, as measured by the 6MWT, were within the expected range of walked distance for age and diseases.

**Table 3:** Anthropometric and biochemical data and walked distance in the 6-min walk test in the study subjects, grouped according to the physical activity program

**Clinical variables**	**Dance program**			**Self-selected program**			**ANOVA**
	**Baseline (n = 42)**	**3 months (n = 41)**	**6 months (n = 41)**	**Baseline (n = 58)**	**3 months (n = 54)**	**6 months (n = 52)**	**P value**
Height (cm)	163 ± 9	- - -	- - -	166 ± 9	- - -	- - -	- - -
Weight (kg)	86.8 ± 17.6	83.4 ± 16.3°	82.9 ± 16.5°	97.3 ± 15.6*	95.0 ± 15.3°*	94.8 ± 15.6°*	0.884
Body mass index (kg/m^2^)	32.7 ± 6.3	31.2 ± 5.1°	31.0 ± 5.1°	35.4 ± 5.8*	34.6 ± 5.4°*	34.5 ± 5.3°*	0.874
Waist circumference (cm)	103 ± 12	99 ± 12°	99 ± 11°	109 ± 10*	107 ± 11°*	107 ± 12°*	0.625
Systolic pressure (mmHg)	131 ± 19	124 ± 15	128 ± 19	128 ± 17	128 ± 17	133 ± 18	0.294
Diastolic pressure (mmHg)	73 ± 3	73 ± 8	73 ± 6	77 ± 9	76 ± 10	76 ± 11	0.705
Glycosylated hemoglobin (%)^	7.46 ± 0.49	7.25 ± 0.73	7.10 ± 0.58	7.46 ± 0.59	7.14 ± 0.77	7.48 ± 0.79	0.011
Fasting glucose (mg/dL)	121 ± 30	115 ± 25°	114 ± 29°	123 ± 31	117 ± 27°	118 ± 29°	0.567
Fasting insulin (μU/mL)	14.2 ± 6.0	12.4 ± 4.8	10.3 ± 2.8	18.5 ± 28.5	17.5 ± 8.1*	17.9 ± 9.4*	0.084
HOMA	3.21 ± 1.81	2.58 ± 1.00	2.11 ± 0.63	4.41 ± 2.33	4.14 ± 2.10*	4.21 ± 2.44*	0.171
Aspartate aminotransferase (U/L)	27.9 ± 11.1	24.9 ± 7.5°	26.8 ± 11.5°	30.8 ± 13.4	27.2 ± 9.6°	28.8 ± 12.7°	0.703
Alanine aminotranferase (U/L)	32.3 ± 11.7	28.4 ± 10.9°	30.8 ± 9.5°	41.7 ± 19.5*	37.5 ± 17.4°*	38.6 ± 18.9°*	0.816
Creatinine (mg/dL)	0.78 ± 0.12	0.77 ± 0.12	0.78 ± 0.11	0.82 ± 0.17	0.82 ± 0.17	0.83 ± 0.16	0.525
Total cholesterol (mg/dL)	192 ± 41	183 ± 35	190 ± 37	198 ± 43	186 ± 44	189 ± 31	0.791
HDL cholesterol (mg/dL)	56.4 ± 15.1	55.5 ± 13.9	58.3 ± 16.4	50.8 ± 13.5	49.7 ± 11.0*	50.9 ± 11.3*	0.275
LDL cholesterol (mg/dL)	112 ± 35	103 ± 30°	110 ± 33	112 ± 38	106 ± 43	115 ± 29	0.940
Triglycerides (mg/dL)	120 ± 71	119 ± 74	108 ± 57	173 ± 109*	149 ± 80	135 ± 52°*	0.275
6-min walk test (m)	415 ± 90	479 ± 56°	499 ± 61°	436 ± 70	460 ± 68°	460 ± 91*	0.015

^Data are limited to the cohort of subjects with diabetes (Dance program, N = 17; Self-selected program, N = 30).

°Significantly different from the corresponding baseline value in the same group.

*Significantly different from the corresponding value in the dance program.

In general, the participation to DP was good (77%), with 34/41 attending >70% of planned sessions. One patient in DP and four in SSAP dropped out before the 3-month control; two patients more were lost in SSAP during the following three months (6-month attrition: DP 2%, SPP 10%, P = 0.233, Fisher’s exact test). Both physical activity programs produced a significant decrease of body weight (−2.6 kg for both; P < 0.001) and waist circumference (DP, −3.2 cm; SSAP, −2.2; P < 0.01) at 3 months, which were maintained at 6 months. Fasting glucose and liver enzymes decreased in both groups. Among biochemical variables, only glycosylated HbA1c showed a different time trend. At 3 months it decreased on average by approximately 0.2-0.3% in the two groups; in the following three months HbA1c decreased by an additional 0.1% in DP, whereas in SSAP it returned to the pre-study values.

No subject experienced hypoglycemic events.

### Physical activity, energy expenditure, and 6-min walk test

**Figure 1:**
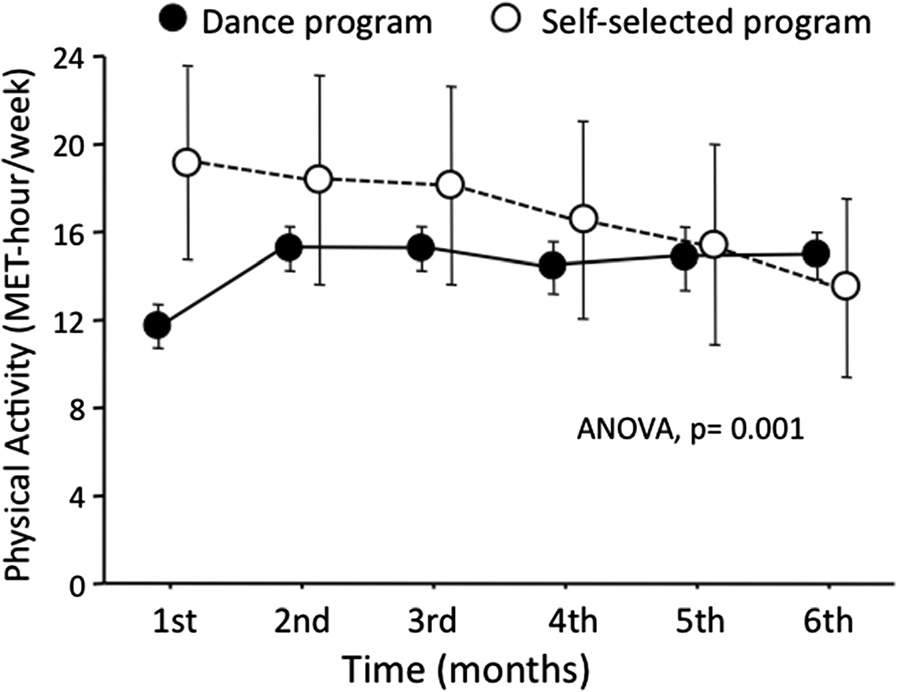
**Time course of activity-related energy expenditure in subjects enrolled in the dance program (black circles) or in the self-selected physical activity program (white circles).** Data are expressed and mean and 95% confidence interval. Note the much wider confidence interval in the self-selected program, expression of very high variability.

The distance at 6MWT increased at 3 months in both groups; after 6 months, the distance further increased by 4% in DP and was unchanged in SSAP. The monthly time course of activity-related energy expenditure was significantly different between groups (ANOVA, P <0.001; Figure 1). In DP it averaged 13.5 ± 1.8 MET-hour/week in the first 3 months and 14.1 ± 3.0 in the second 3-month period. In SSAP it was characterized by a higher and highly variable expenditure in the first 3-month period (16.5 ± 13.9 MET-hour/week), which decreased thereafter to 14.2 ± 12.3 (P vs. first three-month period <0.001).

### Outcome achievement

At 3 months, no differences in target achievement were observed between groups (Table 4). At 6 months the odds ratio of weight loss targets were also not different between groups, whereas the odds to attain the desired 10 MET-hour/week target and 10% increased 6MWT were both significantly associated with DP. At 3 months, the target of 10 MET-hour/week was attained in 39/41 cases (95%) in DP vs. 33/54 (61%) in SSAP (P <0.001; Fisher’s exact test), with 20 cases ≥20 MET-hour/week in SSAP. In the second 3-month period, the target was achieved in 37/41 (77%) in DP vs. 29/52 (56%) in SSAP, where 18 cases exceeded 20 MET-hour/week. Also the odds for the HbA1c target were in favor of DP (not significant); when the cases with prediabetes were added to the analysis, the modest decrease of HbA1c from 7.09 ± 0.65 to 6.84 ± 0.61 in DP (vs. an increase from 7.12 ± 0.73 to 7.18 ± 0.83 in SSAP; ANOVA, P = 0.005) translated into a significant probability to reach the primary HbA1c target (OR, 3.92; 95% confidence interval, 1.06–14.52) in DP.

**Table 4:** Odds ratio to reach the planned targets at three and six month follow-up in the dance program vs. the self-selected activity program

**Primary targets**	**Dance group**	**Self-selected group**	**Odds ratio**	**P value**
	**At target/total cases**	**At target/total cases**	**(95% confidence interval)**	
3-month assessment				
Weight loss (>5%)	12/41	10/54	1.97 (0.63–6.17)	0.246
Reduced waist circumference (>5%)	12/41	13/54	2.02 (0.67–6.06)	0.209
Reduced glycosylated hemoglobin (>0.3%)*	6/17	15/29	0.63 (0.15–2.58)	0.030
Physical activity (>10 MET-hour/week)	39/41	33/54	9.93 (2.00–49.19)	0.005
Increased distance at 6-min walk test (>10%)	20/41	24/54	2.12 (0.77–5.75)	0.146
6-month assessment				
Weight loss (>5%)	10/41	10/52	1.25 (0.38–4.07)	0.716
Reduced waist circumference (>5%)	11/41	11/52	1.27 (0.41–3.99)	0.678
Reduced glycosylated hemoglobin (>0.3%)*	10/17	5/28	6.23 (0.82–47.62)	0.078
Physical activity (>10 MET-hour/week)	37/41	29/52	6.21 (1.70–22.75)	0.006
Increased distance at 6-min walk test (>10%)	28/41	23/52	3.08 (1.12–8.47)	0.029

*Limited to subjects with diabetes.

A separate analysis of target achievement in diabetes (n = 47) and in obesity (n = 53) did not produce qualitatively different results, although in most cases statistical significance was not reached (not reported in details).

## Discussion

The study identifies the benefits of a supervised dance program as part of leisure-time physical activity in improving metabolic control and physical fitness in type 2 diabetes. DP was as effective as SSAP (home, gym, or open-air activity) in the short-term, but the benefits of SSAP were not maintained, with a higher attrition and a progressively reduced energy expenditure along the months, in spite of similar stage-of-change and motivation to physical activity at entry. This underlines the importance of social support and pleasant activities to increase adherence in individual patients, according to cultural and social heritage, translating into significantly better health and psychosocial targets.

The clinical benefits of both activity programs extended from body weight loss to improved glucose control and reduced liver enzymes, to improved physical fitness. The modest but significant reduction of liver enzymes is likely to reflect decreased hepatic triglyceride accumulation [30], as commonly observed in nonalcoholic fatty liver disease entering specific activity programs, also independent of weight loss [31,32]. It should be viewed in the context of the chronic liver disease associated with obesity and diabetes, where intense behavioral treatment [33], also leading to improved glucose control, reduced insulin levels and improved insulin sensitivity [34], is expected to stop liver disease progression [33,35].

The total amount of physical activity was probably underestimated in DP and overestimated in SPP. Most DP patients added physical activity bouts to the two weekly dance sessions, but these events were purposely not recorded to let patients completely free of implementing other activities according to their personal interests, while focusing on strict adherence to DP. On the contrary, all bouts of leisure-time activities were registered in SSAP as primary goal of the study, with a well-known risk of over-reporting compared with objective assessment [36]. Walking, biking and jogging were the most common activities selected in SSAP, with peaks of over 40 MET-hour/week in 7/54 SSAP cases.

During each DP session, the amount of activity was calculated as 4.3 MET-hour on the basis of a repetitive, predetermined Latin and ballroom dance program. According to the Compendium of Physical Activity [20], the energy dance expenditure may vary from 3.5 MET (Caribbean dance, beginners [37]) to >10 MET (Aerobic step and Swedish folk dancing) [38,39]. The program was designed for mature/elderly people, and the theoretical maximum expenditure was limited between 4 and 7 MET [40], to avoid the risk of cardiovascular complications, without differences between genders [41]. This moderate amount was very similar to the amount of supervised aerobic and resistance training tested against physical activity counseling in a large randomized Italian study [12], where exercise significantly improved metabolic parameters. Similarly, 60-min brisk walking three times weekly (approximately 10 MET-hour/week) improved oxygen uptake, lipid and glucose homeostasis and reduced visceral fat [42]. All studies involving gyms, however, need an intense support to limit attrition, whereas the social support of dancing was probably pivotal to keep patients with diabetes on treatment, reducing HbA1c to an extent similar to that achieved in SSAP.

The estimated MET amount was on average >10 MET-hour/week. Di Loreto et al. have shown that physical activity above this target produces metabolic and clinical effects, which become large above the 20 MET-hour/week threshold [11]. This higher amount of physical activity, also associated with reduced costs of pharmacological treatment [11], was more frequently attained in SSAP, where individual cases attained weekly physical activity levels >40 MET-hour/week, but the minimum of 10 MET-hour/week was more common in DP, both during the first and, particularly, during the second 3-month period and the last month when 93% of cases (39/42) in DP met the target vs. 54% in SSAP (28/52; P <0.0001, Fisher’s exact test, in the per-treatment analysis, corresponding to 90% vs. 48% on intention-to-treat).

The study has both strengths and limitations. The main strength is its easy implementation in the community with limited resources and the robust results that might be exported to other typical clinical settings. The limits are the selection of motivated individuals – with minor differences between groups –, the non-randomized nature of the study, the so far time-limited experience, and the methods used to estimate energy expenditure. The selection of subjects after motivational interviewing may be justified by the very low motivation to physical activity commonly observed in individuals with type 2 diabetes [43], also reported in Italian subjects with diabetes [23] as well as other metabolic disorders [24], with a high risk of attrition in unselected patients. The lack of randomization was justified by inherent difficulties in directing individuals to dancing independently of their willingness. To adjust for non-randomization, a propensity score approach was selected. Although less solid than properly planned RCTs, adjusting by propensity produces valuable data reflecting the “real world” of disease treatment [44], and the procedure is largely accepted in chronic diseases requiring motivation as pre-requisite to accept treatment [44]. The study design was chosen to accommodate the patients’ desires to receive their favorite treatment and achieve physical activity benefits while maintaining scientific integrity. In this case, we tried to favor patients by providing a DP, which was also planned according their cultural heritage. In the real setting, every effort should be made to offer motivated patients their preferred activities, as long as they demonstrate long-term effectiveness, and also low-grade activity may be effective [42]. Forcing patients, although motivated, to perform activities largely considered unpleasant in the general population is likely to result in attrition and treatment failure [45]. As to the time-limited experience, the study will be continued and further work is needed to confirm longer effectiveness. Finally, the methods used to estimate energy expenditure has probably led to an underestimation of physical activity in DP and overestimation in SSAP. To overcome this limit, future studies should use more objective devices (i.e., accelerometers, not usually available in standard clinical settings) to assess participants’ expenditure.

In conclusion, dance may be an effective strategy to implement physical activity in motivated subjects with type 2 diabetes or obesity. Following this seminal positive experience, dancing might also be tested as a prevention strategy in cases at high risk of type 2 diabetes in the community, where physical activity may halt or delay disease progression [46]. As a social and pleasant form of exercising, it may be potentially useful, particularly to older and obese patients, also according to cultural and social heritages, to escape isolation and improve quality of life.

## Abbreviations

DP: Dance program; SSAP: Self-selected activity program; MET: Metabolic equivalent; 6-MWT: 6-min walk test; HbA1c: Glycosylated hemoglobin.

## Competing interest

None in relation to the material presented in this report.

## Authors’ contributions

FM, GF, and GM contributed to conception and design of the study. FM and LM collected the data. GF and GM reviewed the literature. RDG and GM wrote the initial draft. All authors substantially contributed to data interpretation and critical revision of the manuscript. All authors read and approved the final manuscript.

## Aknowledgments

The authors are indebted to Mrs. E. Ceccardi, President of Diabetic Association, Brescia Regional area, to nurses E. Neboli and S. Cè, and to Mrs. L. Manuzzato (Organizing Committee) for continuous enthusiastic support.

## Previous presentation

An abstract of the preliminary results of the study was presented at the Annual Meeting of the Associazione Medici Diabetologi (AMD); May 29-June 1, 2013; Rome, Italy.

## References

[B1] American Diabetes Association Executive summary: Standards of medical care in diabetes--2013 Diabetes Care 2013 36 Suppl 1 S4 S10 23264424 10.2337/dc13-S004 PMC3537272

[B2] Montesi L Moscatiello S Malavolti M Marzocchi R Marchesini G Physical activity for the prevention and treatment of metabolic disorders Intern Emerg Med 2013 8 655 666 23657989 10.1007/s11739-013-0953-7

[B3] Knowler WC Fowler SE Hamman RF Christophi CA Hoffman HJ Brenneman AT Brown-Friday JO Goldberg R Venditti E Nathan DM 10-year follow-up of diabetes incidence and weight loss in the Diabetes Prevention Program Outcomes Study Lancet 2009 374 1677 1686 19878986 10.1016/S0140-6736(09)61457-4 PMC3135022

[B4] Colberg SR Sigal RJ Fernhall B Regensteiner JG Blissmer BJ Rubin RR Chasan-Taber L Albright AL Braun B Exercise and type 2 diabetes: the American College of Sports Medicine and the American Diabetes Association: joint position statement Diabetes Care 2010 33 e147 e167 21115758 10.2337/dc10-9990 PMC2992225

[B5] Zhao G Ford ES Li C Balluz LS Physical activity in U.S. older adults with diabetes mellitus: prevalence and correlates of meeting physical activity recommendations J Am Geriatr Soc 2011 59 132 137 21226683 10.1111/j.1532-5415.2010.03236.x

[B6] Ekkekakis P Lind E Exercise does not feel the same when you are overweight: the impact of self-selected and imposed intensity on affect and exertion Int J Obes (Lond) 2006 30 652 660 16130028 10.1038/sj.ijo.0803052

[B7] Helitzer DL Peterson AB Sanders M Thompson J Relationship of stages of change to attendance in a diabetes prevention program Am J Health Prom 2007 21 517 520 10.4278/0890-1171-21.6.517 17674639

[B8] Biddle SJ Fox KR Motivation for physical activity and weight management Int J Obes Relat Metab Disord 1998 22 Suppl 2 S39 S47 9778095

[B9] Di Loreto C Fanelli C Lucidi P Murdolo G De Cicco A Parlanti N Santeusanio F Brunetti P De Feo P Validation of a counseling strategy to promote the adoption and the maintenance of physical activity by type 2 diabetic subjects Diabetes Care 2003 26 404 408 12547870 10.2337/diacare.26.2.404

[B10] The Diabetes Prevention Program Research Group The Diabetes Prevention Program (DPP): description of lifestyle intervention Diabetes Care 2002 25 2165 2171 12453955 10.2337/diacare.25.12.2165 PMC1282458

[B11] Di Loreto C Fanelli C Lucidi P Murdolo G De Cicco A Parlanti N Ranchelli A Fatone C Taglioni C Santeusanio F De Feo P Make your diabetic patients walk: long-term impact of different amounts of physical activity on type 2 diabetes Diabetes Care 2005 28 1295 1302 15920042 10.2337/diacare.28.6.1295

[B12] Balducci S Zanuso S Nicolucci A De Feo P Cavallo S Cardelli P Fallucca S Alessi E Fallucca F Pugliese G Effect of an intensive exercise intervention strategy on modifiable cardiovascular risk factors in subjects with type 2 diabetes mellitus: a randomized controlled trial: the Italian Diabetes and Exercise Study (IDES) Arch Intern Med 2010 170 1794 1803 21059972 10.1001/archinternmed.2010.380

[B13] Fabricatore AN Behavior therapy and cognitive-behavioral therapy of obesity: is there a difference? J Am Diet Assoc 2007 107 92 99 17197276 10.1016/j.jada.2006.10.005

[B14] Ford ES Herman WH Leisure-time physical activity patterns in the U.S. diabetic population. Findings from the 1990 National Health Interview Survey–Health Promotion and Disease Prevention Supplement Diabetes Care 1995 18 27 33 7698044 10.2337/diacare.18.1.27

[B15] Lee RE Mama SK Medina A Orlando Edwards R McNeill L SALSA: SAving Lives Staying Active to Promote Physical Activity and Healthy Eating J Obes 2011 2011 436509 21234315 10.1155/2011/436509 PMC3018638

[B16] Kattenstroth JC Kolankowska I Kalisch T Dinse HR Superior sensory, motor, and cognitive performance in elderly individuals with multi-year dancing activities Front Aging Neurosci 2010 2 pii:31 10.3389/fnagi.2010.00031 PMC2917240 20725636

[B17] Miller WR Rollnick S Motivational Interviewing 2002 2 New York: The Guilford Press

[B18] Associazione Medici Diabetologi, Società Italiana di Diabetologia AMD-SID Cura del diabete Standard Italiani per la Cura del Diabete Mellito 2009–2010 2010 Torino: Infomedica 29 70

[B19] Forlani G Lorusso C Moscatiello S Ridolfi V Melchionda N Di Domizio S Marchesini G Are behavioural approaches feasible and effective in the treatment of type 2 diabetes? A propensity score analysis vs. prescriptive diet Nutr Metab Cardiovasc Dis 2009 19 313 320 18722095 10.1016/j.numecd.2008.06.004

[B20] Ainsworth BE Haskell WL Herrmann SD Meckes N Bassett DR Jr Tudor-Locke C Greer JL Vezina J Whitt-Glover MC Leon AS Compendium of Physical Activities: a second update of codes and MET values Med Sci Sports Exerc 2011 2011 43 1575 1581 21681120 10.1249/MSS.0b013e31821ece12

[B21] Enright PL The six-minute walk test Respir Care 2003 48 783 785 12890299

[B22] Spiller V Scaglia M Meneghini S Vanzo A Assessing motivation to change towards healthy nutrition and regular physical activity. Validation of two sets of instruments Mediterr J Nutr Metab 2009 2 41 47

[B23] Centis E Trento M Dei Cas A Pontiroli AE De Feo P Bruno A Sasdelli AS Arturi F Strollo F Vigili De' Kreutzenberg S Invitti C Di Bonito P Di Mauro M Pugliese G Molteni A Marchesini G Stage of change and motivation to healthy diet and habitual physical activity in type 2 diabetes Acta Diabetol 2014 e-Pub Jan 20 10.1007/s00592-013-0551-1 24442514

[B24] Centis E Moscatiello S Bugianesi E Bellentani S Fracanzani AL Calugi S Petta S Dalle Grave R Marchesini G Stage of change and motivation to healthier lifestyle in non-alcoholic fatty liver disease J Hepatol 2013 58 771 777 23201248 10.1016/j.jhep.2012.11.031

[B25] Prochaska JO Velicer WF The transtheoretical model of health behavior change Am J Health Promot 1997 12 38 48 10170434 10.4278/0890-1171-12.1.38

[B26] Miller WR Motivational interviewing with problem drinkers Behav Psychother 1983 11 147 172

[B27] Bandura A Self-efficacy: toward a unifying theory of behavioral change Psychol Rev 1977 84 191 215 847061 10.1037//0033-295x.84.2.191

[B28] Rubin DB The design versus the analysis of observational studies for causal effects: parallels with the design of randomized trials Stat Med 2007 26 20 36 17072897 10.1002/sim.2739

[B29] Duncan DB Multiple range test for correlated and heteroscedastic means Biometrics 1957 13 164 204

[B30] Johnson NA Keating SE George J Exercise and the liver: implications for therapy in fatty liver disorders Semin Liver Dis 2012 32 65 79 22418889 10.1055/s-0032-1306427

[B31] St George A Bauman A Johnston A Farrell G Chey T George J Independent effects of physical activity in patients with nonalcoholic fatty liver disease Hepatology 2009 50 68 76 19444870 10.1002/hep.22940

[B32] Montesi L Caselli C Centis E Nuccitelli C Moscatiello S Suppini A Marchesini G Clinical Audit: Physical activity support or weight loss counseling for nonalcoholic fatty liver disease? World J Gastroenterol 2014 in press 10.3748/wjg.v20.i29.10128 PMC4123342 25110440

[B33] Moscatiello S Di Luzio R Bugianesi E Suppini A Hickman I Di Domizio S Dalle Grave R Marchesini G Cognitive-behavioral treatment of non-alcoholic fatty liver disease: a propensity score-adjusted observational study Obesity (Silver Spring) 2011 19 763 770 20966900 10.1038/oby.2010.254

[B34] Moscatiello S Di Luzio R Sasdelli AS Marchesini G Managing the combination of nonalcoholic fatty liver disease and metabolic syndrome Expert Opin Pharmacother 2011 12 2657 2672 22043839 10.1517/14656566.2011.629188

[B35] Promrat K Kleiner DE Niemeier HM Jackvony E Kearns M Wands JR Fava JL Wing RR Randomized controlled trial testing the effects of weight loss on nonalcoholic steatohepatitis Hepatology 2010 51 121 129 19827166 10.1002/hep.23276 PMC2799538

[B36] Schmidt MD Freedson PS Chasan-Taber L Estimating physical activity using the CSA accelerometer and a physical activity log Med Sci Sports Exerc 2003 35 1605 1611 12972884 10.1249/01.MSS.0000084421.97661.17

[B37] Di Blasio A De Sanctis M Gallina S Ripari P Are physiological characteristics of Caribbean dance useful for health? J Sports Med Phys Fitness 2009 49 30 34 19188893

[B38] Olson MS Williford HN Blessing DL Greathouse R The cardiovascular and metabolic effects of bench stepping exercise in females Med Sci Sports Exerc 1991 23 1311 1317 1766349

[B39] Wigaeus E Kilbom A Physical demands during folk dancing Eur J Appl Physiol Occup Physiol 1980 45 177 183 7193127 10.1007/BF00421325

[B40] Vivarelli C Chiarandini G Zadro I Antonutto G Tuniz D Estimated energy expenditure in elderly subjects during ballroom dancing Med Sport 2008 61 429 442

[B41] Blanksby BA Reidy PW Heart rate and estimated energy expenditure during ballroom dancing Br J Sports Med 1988 22 57 60 3167503 10.1136/bjsm.22.2.57 PMC1478556

[B42] Herzig KH Ahola R Leppaluoto J Jokelainen J Jamsa T Keinanen-Kiukaanniemi S Light physical activity determined by a motion sensor decreases insulin resistance, improves lipid homeostasis and reduces visceral fat in high-risk subjects: PreDiabEx study RCT Int J Obes (Lond) 2013 e-Pub Nov 28 10.1038/ijo.2013.224 PMC4125749 24285336

[B43] Vähäsarja K Salmela S Villberg J Rintala P Vanhala M Saaristo T Peltonen M Keinänen-Kiukaanniemi S Korpi-Hyövälti E Kujala UM Moilanen L Niskanen L Oksa H Poskiparta M Perceived need to increase physical activity levels among adults at high risk of type 2 diabetes: a cross-sectional analysis within a community-based diabetes prevention project FIN-D2D BMC Public Health 2012 12 514 22781026 10.1186/1471-2458-12-514 PMC3506518

[B44] D'Agostino RB Jr Propensity scores in cardiovascular research Circulation 2007 115 2340 2343 17470708 10.1161/CIRCULATIONAHA.105.594952

[B45] Hibbard JH Tusler M Assessing activation stage and employing a "next steps" approach to supporting patient self-management J Ambul Care Manage 2007 30 2 8 17170632 10.1097/00004479-200701000-00002

[B46] Ma J Yank V Xiao L Lavori PW Wilson SR Rosas LG Stafford RS Translating the Diabetes Prevention Program lifestyle intervention for weight loss into primary care: a randomized trial Arch Intern Med 2012 9 1 9 10.1001/2013.jamainternmed.987 PMC3856315 23229846

